# There ain't nothing like a Dame: a commentary on Lonsdale (1947) ‘Divergent beam X-ray photography of crystals’

**DOI:** 10.1098/rsta.2014.0232

**Published:** 2015-04-13

**Authors:** A. M. Glazer

**Affiliations:** Clarendon Laboratory, Parks Road, Oxford OX1 3PU, UK

**Keywords:** X-ray diffraction, divergent beam, Kossel patterns, diamond

## Abstract

Prof. Dame Kathleen Lonsdale was one of the two first female Fellows of the Royal Society, having originally been a student of that great British scientist and Nobel Laureate William Henry Bragg. She came to fame initially for her solution of the crystal structure of hexamethyl benzene, thus demonstrating that the benzene ring was flat, of considerable importance to organic chemistry, where it had been proposed before but without proof. This was at a time when the solution of crystal structures was in its infancy, and in its day this work was considered a triumph. As a rare example then of a female physicist, Lonsdale became interested in various aspects of the diffraction of X-rays, and in particular published an important paper on a form of diffraction in which a strongly divergent source was used rather than the usual highly collimated beam. The photographs thus obtained showed a series of arcs and circles, whose positions were so sensitive that they could be used to determine the quality of crystals such as diamond, and even to calculate their lattice dimensions, and hence carbon–carbon bond lengths, to hitherto extraordinary precision. Lonsdale also became known not just as a scientist but as a peace activist and an active member of the Society of Friends. This commentary was written to celebrate the 350th anniversary of the journal *Philosophical Transactions of the Royal Society*.

## 1. Kathleen Lonsdale

**Figure 1.**
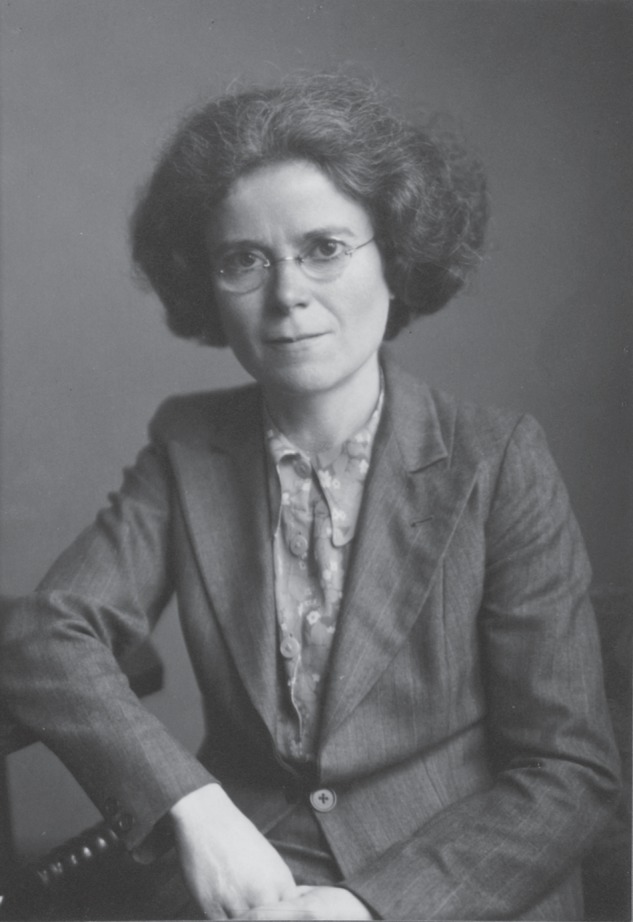
Kathleen Lonsdale *ca* 1935. Copyright The Royal Society.

Kathleen Lonsdale (née Yardley) (figure 1) was born in Newbridge, Ireland, on 28 January 1903 and died 1 April 1971 in London. A full account of her life and work was written by Dorothy Hodgkin for the Royal Society [1] in 1975. Here, I shall give a brief summary as well as some personal impressions.

Kathleen studied physics at Bedford College for Women in London, graduating top of her year in 1922. This achievement brought her to the attention of William Henry Bragg (hereafter, William Bragg) at University College London (UCL), who in 1912 worked with his son William Lawrence Bragg (hereafter, Lawrence Bragg) to found the then new discipline of X-ray crystallography (see below). Both father and son shared the 1915 Nobel Prize in Physics for their work and both established active research groups after World War I. Lawrence Bragg set up his group at Manchester University, concentrating mainly on metals and inorganic crystals, while his father went to University College London, concentrating mainly on organic crystals, in order to avoid too much overlap with his son's work.

William offered Kathleen a place in his research team (it is interesting to note that both Braggs were active in encouraging women into scientific research, and at a time when such enlightenment was rare. Indeed, William had 18 research students among whom 11 were women!). In 1923, William moved to the Royal Institution of Great Britain, taking Kathleen Lonsdale with him.

An important contribution came in 1924 out of a collaboration with William T. Astbury to produce the famous Astbury and Yardley tables entitled ‘Tabulated data for the examination of the 230 space groups by homogeneous X-rays’ [2] and submitted by W. H. Bragg. Theoretical work by A. Schoenflies, E. Federov and W. Barlow in the 1890s [3–5] had shown that there are only 230 possible spatial symmetries that all crystals could adopt (apart from certain more exotic types such as the so-called incommensurate and quasi-crystals, discovered many years later). Astbury and Yardley's tables described all of the space groups in a form that enabled crystallographers to use space group symmetry as part of the process of determining crystal structures. In later years, these tables formed the basis for the International Tables for Crystallography which have now become the standard reference on space group symmetry for all crystallographers today.

Following this she solved two very important crystal structures, those of hexamethyl benzene [6] and hexachlorobenzene [7]. The importance of this work was that it proved for the first time something that organic chemists had been conjecturing for some time, but had no sure evidence for, namely that the benzene ring is flat, rather than puckered. This proof then opened up a proper understanding of the whole of aromatic organic chemistry. At the time, of course, there were no computers, and so to derive a crystal structure like this with any degree of confidence was a major triumph.

In 1936, Kathleen [8] published a book, written entirely in her neat handwriting, entitled ‘Simplified structure factor and electron density formulae for the 230 space groups of mathematical crystallography’ on behalf of the Royal Institution. This gave the mathematical relationships between possible X-ray diffraction amplitudes according to the space group symmetry of the crystal. Before the age of computers this mammoth work was of great importance in helping to solve crystal structures.

In those days, X-ray apparatus was very crude compared with today's laboratory X-ray tubes and generators, and especially so in comparison with modern synchrotron sources. Kathleen Lonsdale became adept at constructing her own apparatus, often at risk to her own health. It was difficult to have a stable X-ray source for more than an hour or so. Final lining up of an X-ray camera was sometimes achieved by looking directly by eye through the collimator at the glowing filament of the X-ray tube! She was also accomplished in carrying out experiments under different conditions, such as at low temperature. W. Hume-Rothery [9] remembered a visit to the Royal Institution where he saw ‘the figure of Dr. Lonsdale appearing through a cloud of mist, like a glorified spectre of the Brocken, while her assistant pumped liquid air over a crystal’.

In 1949 she took up the post of Professor of Chemistry at UCL and established a large and active crystallographic research group, retiring finally in 1968. Kathleen and members of her research group studied a variety of topics, including magnetic anisotropy of crystals, crystal symmetry, X-ray diffuse scattering (arising from elements of disorder in crystals), and towards the end of her scientific career, chemical reactions in crystals and the crystallography of bladder stones.

On the personal side, Kathleen was a convinced Quaker and was active in peace movements. During the Second World War she famously refused to register for employment and civil defence duties because of her conscientious objection. After refusing to pay a £2 fine she was sent to Holloway Prison for one month. Following this she took an active interest in prison reform and often visited prisoners, even succeeding in visiting prisons in the Soviet Union.

Perhaps I may be allowed here to inject some personal reminiscences of Kathleen Lonsdale. Together with H. D. Flack, I was one of her two last research students before she retired in 1968. In February 1965, while I was finishing my degree in chemistry in St Andrews University, Kathleen invited me to University College London to meet her in order to discuss taking up a studentship with her. I recall arriving in the rather dingy and poorly lit basement of the Chemistry Department at UCL to meet her for the first time. I was struck with how tiny she seemed, extremely thin (I later saw that she ate very little: a few lettuce leaves, some nuts, no alcohol!) with a shock of white frizzy hair. She looked as if a feather could knock her over. However, as I was to learn later, despite her appearance she was very determined and tough, and did not suffer fools gladly. Nonetheless she was charming and welcoming. She showed me around the research area and I recall seeing many posters on the walls including some strange photographs with curved lines. I later realized much later that this was her work on divergent beam diffraction, the subject of this article.

When I started in the group in the following October, Kathleen suggested that I should work on mixed organic crystals, but it was not until the following June that I was able to find the system in which I continued my research. From time to time she would summon one of us upstairs to her office to discuss progress. On arriving in her office the first question was always the same: where are the Laue photos (these are X-ray photographs taken of a stationary crystal using an unfiltered X-ray beam of ‘white’ radiation, i.e. a continuum of wavelengths rather than with a single fixed wavelength)? And if we had failed to bring them with us, we were in serious trouble! She had a gift in interpreting Laue photographs and was able to spot features that most of us had ignored. Our training in crystallography was very classical, for which I have remained ever grateful. It meant that we learnt all the basic theory and techniques used by crystallographers, such as how to orient crystals in an X-ray beam, an art that today has largely been forgotten. Some of these techniques were never published but handed down by word of mouth. I remember that early on she made us learn to use Beevers–Lipson strips by calculating a simple electron density map of hexamethylbenzene. These strips consisted of hundreds of strips of paper with numbers on them and were used before the age of computers to add together many sine and cosine terms in order to draw maps of the distribution of electrons in a crystal structure. After a week's solid work we came out with a very crude map, thus illustrating clearly how difficult crystallography had been earlier on. A great learning experience! We did have a computer in the group, a Ferranti Pegasus Mark II, which had been given to Kathleen. I think we were just about the only group to have such a machine of our own for research purposes. Although incredibly slow and difficult to use by modern standards, this machine with 8 K of store on magnetic drums enabled us to perform most of our calculations, and was a far cry from Beevers–Lipson strips. Refinement of the atomic positions in a crystal was made by feeding in 5-hole paper tape and then recycling the output tape back in again in order to iterate to the solution. This actual computer can now be seen in the Science Museum of London, where it is the oldest working electronic valve computer in the world. Intensities of X-ray reflections were then measured mainly on films by eye, by comparing spot intensities with a prepared scale, a most tedious process, and simple calculations when not using Pegasus were made with a FACIT mechanical calculator.

## 2. Divergent beam diffraction

In 1947, Kathleen Lonsdale published a major paper entitled ‘Divergent beam X-ray photography of crystals’ [10]. In order to understand this work and its importance, it is interesting to go back briefly to the first demonstration of the diffraction of X-rays by Max Laue, Paul Knipping and Walter Friedrich in the spring of 1912.

Earlier in 1895, Wilhelm Roentgen had accidentally discovered some mysterious radiation that could penetrate through solid material. It was soon established that these rays were not affected by magnetic or electric fields and so the question arose as to what they were. At the time, the burning question about X-rays was whether they consisted of waves or beams of particles. William Bragg, at the time working in Adelaide, had been engaged on the study of alpha particles and became interested in this problem. Based on several experimental observations, he became convinced that X-rays (as they had by then become known) consisted of neutral particles, whereas it is fair to say that most physicists disagreed, instead believing them to have wave-like properties. The argument was intense, often bitter and sometimes led to angry exchanges. In 1912, this question was under consideration by Arnold Sommerfeld's group in Munich, where Max Laue had been taken on as *Privatdozent*. According to the normally accepted story, it was during a conversation with one of Sommerfeld's students, Paul Ewald, that Laue had the brilliant idea that if crystals consisted of a regular array of atoms whose spacings were in the ångstrom region, it might be possible to diffract X-rays, thus demonstrating their wave-like properties. So well and so good.

However, the story is tempered by an assumption that was made by Laue, namely that the incident X-rays would stimulate secondary radiation by certain atoms within the crystal and it would be these X-rays that would be diffracted by the surrounding atoms (this was the reason why the first crystal to be examined was that of copper sulfate, as the copper atom was known to strongly fluoresce to produce secondary X-rays. In fact, it was a most unsuitable crystal to begin with as its low symmetry meant that any interpretation of the results at the time would have been unfeasible). This would mean that the diffracting X-rays would consist of a single wavelength. Initially Sommerfeld turned down Laue's request to use the Institute's personnel to carry out the experiment, and so Laue had to resort to having this done in secret (Ewald once told me that Friedrich and Knipping actually had to steal Roentgen's own X-ray apparatus in order to carry out this trial experiment). Following the first successful demonstration of X-ray diffraction, it is reported that Sommerfeld threw a celebratory party, but failed to invite Laue! Such was the atmosphere in the institute at that time. The successful observation of a diffraction pattern appeared to support the wave theory for X-rays. However, Laue, who was later awarded the 1914 Nobel Prize in Physics, had great difficulty in explaining the pattern of spots obtained, because he was inexplicably wedded to the idea of fluorescent X-rays being the cause of the diffracted radiation. This was despite the fact that Friedrich and Knipping had obtained perfectly good diffraction patterns from a crystal of diamond, where fluorescence would not have been expected: in retrospect, it is surprising that Laue did not realize that this obviously demonstrated that the diffraction was not due to secondary radiation at all. Moreover, in trying to explain all the spots, and those that were in fact missing, Laue had also assumed incorrectly that, for instance, in the case of ZnS, the molecules were situated at the corners of a cube and that up to five distinct wavelengths might be involved.

It was later that year that the 22 year old Lawrence Bragg showed how to explain the diffraction patterns in terms of reflection from planes of ZnS molecules occurring in a face-centred cubic array, and more importantly from the point of view of the present discussion that the diffraction occurred using an incident white (continuous wavelengths) X-ray beam. His famous Law *n*λ = 2*d* sin *θ* requires that three quantities, wavelength, spacing and angle be such that this equation is satisfied, and only then does diffraction occur (incidentally Lawrence Bragg initially wrote this formula in its cosine form, but it was subsequently changed to the accepted sine form after working with his father William Bragg's ionization spectrometer. I believe that this change was because in a spectrometer it is natural to measure angles of deflection from a zero position defined by the incident beam and this was 90° from Lawrence Bragg's initial choice of angles). Interestingly as late as 1913 Laue was still thinking that a white incident beam would cause uniform fogging of the X-ray film, whereas it is easy to see that Bragg's Law picks out from a white beam only those wavelengths that satisfy the equation. It is an interesting thought to consider that had a single wavelength really been involved as originally thought by Laue, then Bragg's Law shows that with a stationary crystal it is highly unlikely that it would have been satisfied and X-ray diffraction would not have been discovered when it was. Laue would not have received his Nobel Prize, nor probably would the Braggs in 1915!

Now the reason why I have mentioned this early work of Laue is because in fact, to his credit, he was not completely wrong in his assertion that secondary radiation was, or better could be, involved. It is just that the techniques available at the time would not have enabled this to be observed. An excellent account of the history of this period in the field of crystallography can be read in the book by Authier [11].

It was in 1914 that William Bragg first observed, using his ionization spectrometer, that when a beam of X-rays was selectively reflected from a crystal of diamond, there was a small amount of absorption of the radiation transmitted at the reflection angle. While most experiments used a beam of X-rays that was reasonably parallel, Rutherford & Andrade [12] observed both reflection and absorption, using a point source of divergent *γ*-rays emanating from radium with a crystal of rock salt. This enabled them to make a precise measurement of the wavelength of the monochromatic radiation. Between 1916 and 1917, in a series of papers, Seemann [13,14] used a specially constructed X-ray tube that gave a wide-angle beam of radiation from a copper target diffracted in the back-reflection position from a crystal of rock salt. This gave a symmetrical pattern of reflection conics, as opposed to the spots seen with a parallel beam of X-rays. Such cone-like patterns were also seen later in electron diffraction images by Kikuchi [15,16].

Up to 1935, the divergent beam work had been conducted with the crystal outside the X-ray tube. Kossel & Voges [17] in 1935 showed that similar effects could be obtained by using a single crystal containing a fluorescent substance as the target material in an X-ray tube (X-rays are produced when a beam of electrons strike a metal target), whereupon a strongly divergent beam of X-rays is created within the crystal. The arrangement of conics today is normally called a Kossel pattern. Borrmann [18] also used fluorescent X-rays from a single crystal as his divergent beam and observed both reflection conics and Laue spots. It was also shown by Fujiwara [19,20] and Onoyama [21] that similar effects could be had using a convergent rather than divergent beam.

**Figure 2.**
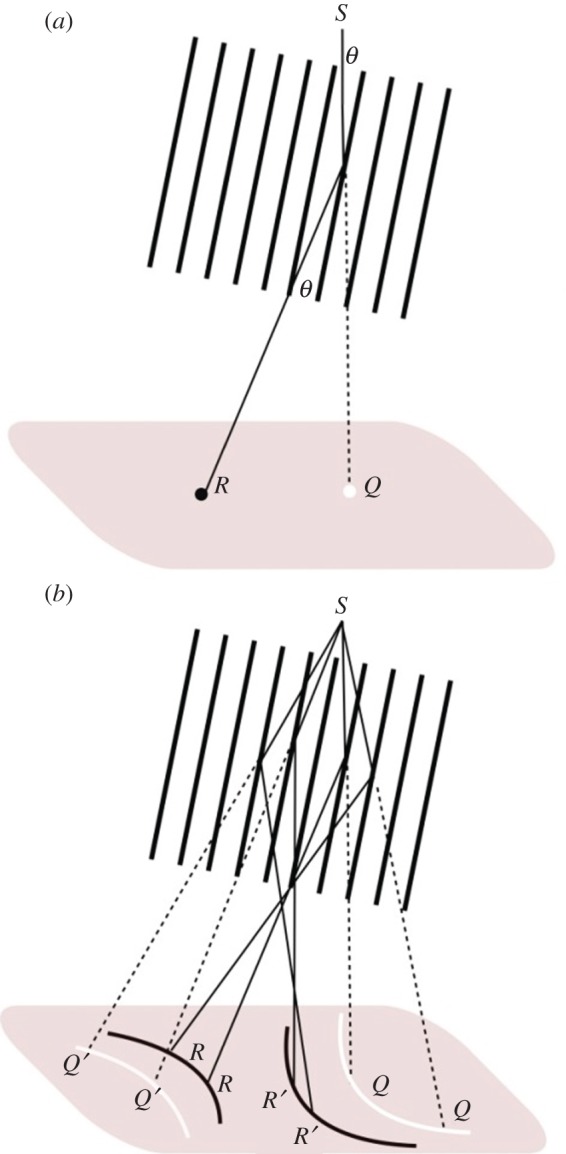
Formation of black and white conics by reflection and absorption of rays (diagrams adapted from fig. 2 of Lonsdale [10]).

To understand the origin of the conics seen in the divergent beam photographs, start with figure 2*a*. *S* is a point source of X-rays and a ray from this point passes through the set of planes in the crystal at an angle *θ* to the set of planes to arrive on the photographic plate at the position marked *Q*. Since this ray has passed through the crystal undeviated, its intensity will be slightly diminished by absorption. This will then appear as a light spot against the general background (the background radiation is caused by many different contributions, including scattering by the air and diffuse scattering due to any disorder of the atomic arrangement in the crystal structure). The beam will also be reflected through the equivalent angle according to Bragg's Law to end up at the position *R* as a dark spot. Figure 2*b* shows four beams passing through and being reflected by the planes. Note that corresponding to the darkened positions marked *R* there are equivalent lighter positions marked *Q*, and similarly for *R*′ and *Q*′. Generalizing this, we see that a series of arcs is created with lighter arcs on the outside and darker arcs on the inside. Thus, the light arcs correspond to where absorption of the X-rays occurs, whereas the dark arcs correspond to the X-ray reflection from the planes.

**Figure 3.**
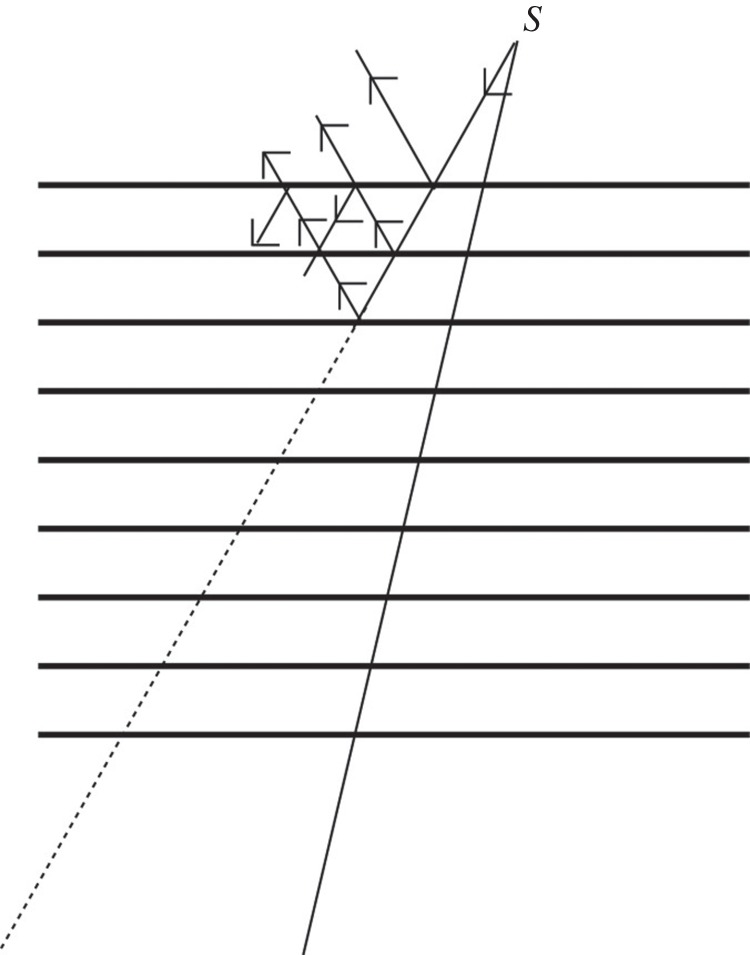
Reflection and transmission by a perfect crystal (adapted from fig. 3 of Lonsdale [10]).

One of the problems associated with divergent beam diffraction is to do with crystal perfection. Rutherford and Andrade, for instance, reported that very perfect crystals of rock salt gave much inferior results to crystals of lesser perfection. This effect is well known to crystallographers as *extinction* and as such is usually avoided wherever possible when using crystals for structure determination. Suppose the crystal is perfect and thus all the atomic planes are perfectly parallel to one another (figure 3). In this case, a ray at the Bragg angle from the source point *S* is partially reflected by the first plane with most of the intensity passing through to the second plane. Scattering then takes place by the second plane. However, this scattered beam meets the first plane at the same Bragg angle and then a fraction of its intensity is scattered back into the forward direction. This leads to a series of multiple reflections. Because of this, divergent beam photographs of such crystals using a fine divergent source of X-rays tend to give such fine lines that they cannot be seen.

**Figure 4.**
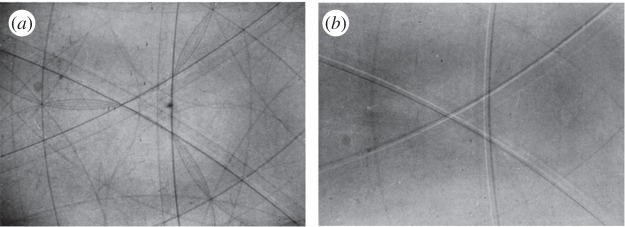
(*a*,*b*)Two examples of divergent beam photographs of diamond (figs 22*a* and *c* from Lonsdale [10] (Copyright The Royal Society)).

In order to obtain her divergent beam photographs, Kathleen borrowed a special X-ray tube from a colleague, Alex Müller, at the Royal Institution. This had been designed to provide a beam of copper radiation divergent through an angle of about 180°. With this tube, she was able to obtain excellent photographs with unusually short exposure times of about 3 s at a distance of 6 cm using the kind of X-ray sensitive film available at the time. The crystals used were mainly of diamond in Lonsdale's paper [10], although some experiments were mentioned for organic crystals, and even on natural ice crystals. The crystal was placed very close to the X-ray tube in order to make use of the highest divergence of the beam as possible. The experimental arrangement therefore was very simple, but the main strength of Lonsdale's paper lies in her ability to use the divergent beam photographs to calculate the most precise values for the unit cell lattice constants and hence the carbon–carbon bond lengths in diamond. This precision has hardly been superseded since. Some examples of her photographs are shown in figure 4. Figure 4*a* is a photograph from a crystal described as a Type I diamond with a green skin (diamonds are found in two types, I and II—Type I diamonds show extra reflections in X-ray diffraction photographs using white radiation). The conics can clearly be seen including both absorption and reflection contrast. Figure 4*b* is from another diamond which has been aligned along a threefold axis of symmetry. Here again the conics are obvious, although in this case only some of the lines show the corresponding absorption conics. Note also that the conics occur in pairs with one component much weaker than the other. This arises from the fact that the X-rays used consisted of two close wavelengths, referred to as *Kα*_1_ and *Kα*_2_, rather than a single wavelength.

The real importance of such photographs is in the fact that very high sensitivity in the study of lattice parameters can be obtained especially from the places where the conics cross, giving well-defined points of intersection. The distances between intersections can be measured very precisely and are very sensitive to small changes in the lattice dimensions. They are also insensitive to modest misalignments of the sample.

**Figure 5.**
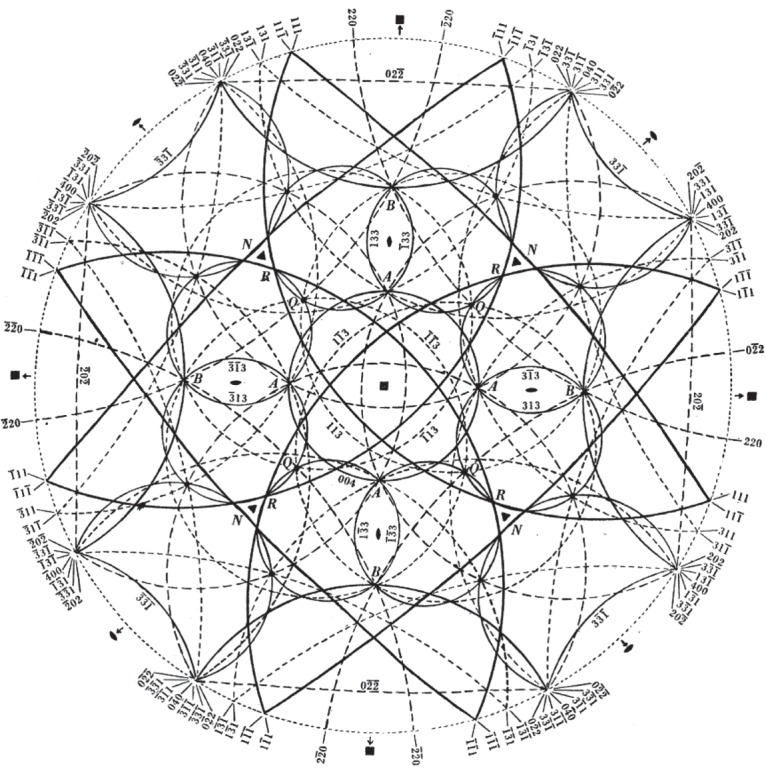
Stereographic projection observed lines down the fourfold axis of a diamond crystal using copper *Kα*_1_ radiation (fig. 12 of Lonsdale [10] (Copyright The Royal Society)).

**Figure 6.**
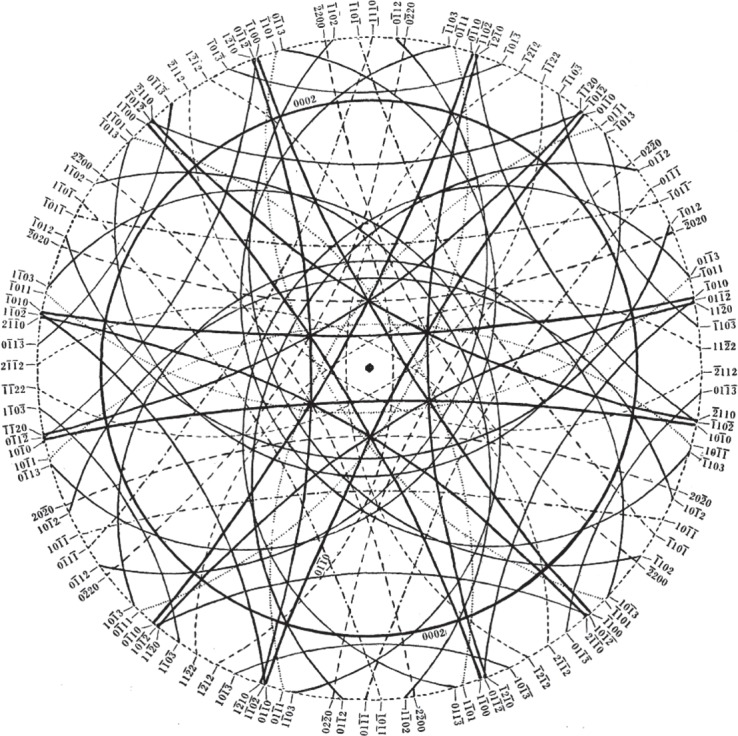
Stereographic projection for natural ice at 0°C (fig. 14 of Lonsdale [10] (Copyright The Royal Society)).

In order to find the lattice dimensions, Lonsdale had first to determine which planes gave rise to particular conics. Fortunately for a crystal such as diamond, which has cubic symmetry, this was relatively easy to do, especially as computers were not available at the time Lonsdale carried out these experiments. Nonetheless her analysis was careful and intricate, obviously requiring a great deal of time to assemble. Today, more complex crystals could be analysed in divergent beam mode through the use of modern computing power. As examples of Kathleen Lonsdale's ability to analyse complex photographs of this kind, see figure 5, where the conics are drawn in a special type of projection known as a stereographic projection (think of this as being like a view of the earth looking down on the North Pole). The numbers marked on the diagram are the so-called Miller indices which denote the planes that give rise to each conic. Note the fourfold symmetry of this diagram. Also note the arrangement of lines around the points marked *R* and compare with figure 4*b*. Figure 6 is a stereographic projection for natural ice showing the arrangement of lines around the sixfold axis of symmetry.

In order to measure lattice parameter values, Lonsdale looked for intersections or overlaps of various conics and used geometry to determine values of the unit cell length assuming knowledge of the wavelength. Another method that she employed was to make use of the close wavelength doublet, *Kα*_1_ and *Kα*_2_. From the distances between the doubled conics, she could then obtain very precise measurements without the need for any particular precision apparatus. Examples of the precision of her measurements for nine different diamonds are given in table 1.

**Table 1.** Measurements made for nine different diamonds.

lattice constant in Å	C−C in Å
3.56723(5)	1.54465(2)
3.56707(2)	1.54458(2)
3.56681(5)	1.54447(2)
3.56665(10)	1.54440(4)
3.56695(5)	1.54453(2)
3.56685(5)	1.54449(2)
3.56671(10)	1.54443(4)
3.5669(1)	1.5445(1)
3.5669(10)	1.5445(4)

From this, Lonsdale estimated that the carbon–carbon distance is 1.54451 Å with a variation of up to ±0.00014 Åin different diamonds. These variations were almost certainly caused by impurities. Therefore, one of the important potential uses of the divergent beam technique is in the study of crystal quality and the effect of imperfections and strain.

In a further use of the technique, Lonsdale went on to use an X-ray target made of brass. This meant that lines from copper and zinc radiations were superimposed on the film. Then by measuring the distances between the conics she was able to find the zinc X-ray wavelength to six significant figures with reference to that of copper. She did raise the question as to whether the wavelengths of copper and zinc X-rays from brass would be identical with those from pure metals, but felt that there was no theoretical reason for a measurable difference.

Lonsdale sent her paper for publication in June 1945 and after referees' reports it was read in February 1946, and finally published in 1947 [10]. The referees were William (Bill) Astbury and Lawrence Bragg. Both referees declared the work as excellent. Bragg, in particular, wrote ‘I recommend the Transactions because this paper is long and contains many illustrations. The length is due to the very detailed way in which the author goes into the various points. It is excellent work, careful and thorough, and is a new method of getting a highly accurate relationship between crystal spacing and say wavelength’. Astbury commented ‘This is an excellent paper and a valuable addition to the literature of X-ray analysis’.

## 3. Later developments

Following publication of Kathleen's landmark paper the subject of divergent beam X-ray diffraction was largely ignored outside of Germany, except perhaps in the field of electron diffraction where this is a commonly observed effect (known as Kikuchi lines, they are formed in electron diffraction patterns by diffusely scattered electrons, e.g. as a result of thermal atom vibrations). However an ingenious suggestion by H. J. Grenville-Wells (later Milledge) working in Kathleen Lonsdale's group was published [22] in 1951 as a way to produce a highly monochromatic but divergent beam in order to improve quantitative measurements. This was to be accomplished by passing a white divergent X-ray beam into a spherical cavity of copper with a pin-hole on the position directly opposite to the incident beam entry point. I am unaware, though, of whether such a device has actually ever been constructed.

With the availability of synchrotron sources in the 1970s producing high-intensity X-radiation, studies of divergent beams have begun to be reinvestigated as a means of studying imperfections in crystals. For instance, a demonstration of divergent beam conics was made using synchrotron radiation by Ullrich *et al*. [23] in 1994 using crystals of copper and gallium arsenide, whose atoms naturally fluoresced in the X-ray beam. In this case, a white beam of incident X-rays was employed. The X-rays produced by a synchrotron source are highly parallel, but the resulting fluorescent X-rays are strongly divergent from within the crystal and so act just like the divergent beams from an ordinary X-ray tube. Schetelich *et al*. [24] in the following year carried out similar experiments using a monochromatic beam to gain resolution. Langer *et al*. [25] in 2001 then showed that, by using an electron beam exciting fluorescence in a crystal of iron, conics could be photographed using a special high-resolution CCD camera, and with this to obtain a precise measurement of the lattice constant. All these methods of course only work for crystals containing X-ray fluorescent atoms.

As a way round this limitation, in 2004 we used the white beam from a synchrotron incident on a crystal of diamond on the surface of which had been stuck a metal foil of niobium. The niobium foil produced fluorescent X-rays and thus acted as a divergent X-ray source at the surface of the diamond [26]. This last experiment was really a modern version of the experiments carried out by Lonsdale in 1945 [10]. In respect of the future use of this type of diffraction effect, it has to be said that it is a specialized technique that would be of use mainly within materials science. With the use of modern computers, it is now possible to analyse routinely the divergent beam patterns even for crystals of low symmetry, and this opens up the range of crystals that can be examined. A good review of the field has been given by Lider [27].

## Author profile

**Mike Glazer** is Emeritus Professor of Physics at the University of Oxford and Visiting Professor at the University of Warwick. He is also Vice-President of the International Union of Crystallography. His PhD research between 1965 and 1968 was under the supervision of Kathleen Lonsdale at University College London, working on the crystallography of organic mixed crystals. In 1968–1969, he was a Fellow at Harvard University, and then from 1969 to 1976 he was at the Cavendish Laboratory, Cambridge. In 1976, he was appointed Lecturer in Physics at the Clarendon Laboratory Oxford and as an Official Fellow and Tutor at Jesus College Oxford. Mike Glazer's research has mainly been in understanding the relationship between physical properties of crystals and their structures. He is perhaps best known for his classification system for tilted octahedra in perovskites. He is also one of the co-founders of Oxford Cryosystems Ltd, which supplies the world market in low-temperature apparatus for crystallographers.
